# Outcomes of general anesthesia versus conscious sedation for Stroke undergoing endovascular treatment: a meta-analysis

**DOI:** 10.1186/s12871-019-0741-7

**Published:** 2019-05-10

**Authors:** Teng-Fei Wan, Rui Xu, Zi-Ai Zhao, Yan Lv, Hui-Sheng Chen, Liang Liu

**Affiliations:** 1 Department of First Cadre Ward, the General Hospital of Northern Theater Command, No. 83 Wenhua Street, Shenyang, 110016 Liaoning China; 2 Department of Neurology, Xinqiao Hospital, the Army Medical University, NO. 183 Xinqiao mian street, Chongqing, 400037 China; 3 Department of Neurology, the General Hospital of Northern Theater Command , No. 83 Wenhua Street, Shenyang, 110016 Liaoning China

**Keywords:** Ischemic stroke, Anesthesia, Endovascular treatment, Meta-analysis

## Abstract

**Background:**

The impact of anesthesia strategy on the outcomes of acute ischemic stroke (AIS) patients undergoing endovascular treatment is currently controversy. Thus, we performed this meta-analysis to compare the differences of clinical and angiographic outcomes between general anesthesia (GA) and conscious sedation (CS).

**Methods:**

A literature search in PubMed, Embase, and Web of Knowledge databases through February 2019 was conducted for related records on GA and CS of AIS undergoing endovascular treatment. The results of the studies were pooled and meta-analyzed with fixed- or random-effect model based on heterogeneity test in total and subgroup analyses.

**Results:**

Twenty-three studies including 6703 patients were analyzed in this meta-analysis. We found that patients in the GA group have lower odds of favorable functional outcome (mRS scores ≤2) compared with the CS group (odds ratio [OR] = 0.62, 95% confidence interval [CI]: 0.49–0.77), and higher risk of mortality (OR = 1.68, 95% CI: 1.49–1.90), pneumonia (OR = 1.78, 95% CI: 1.40–2.26), symptomatic intracranial hemorrhage (OR = 1.64, 95% CI: 1.13–2.37). However, no significant differences were seen between the groups in the rate of recanalization (OR = 1.07, 95% CI: 0.89–1.28), vessel dissection or perforation (OR = 1.00, 95% CI: 0.98–1.03) and asymptomatic intracranial hemorrhage (OR = 1.19, 95% CI: 0.96–1.47). While in the RCT subgroup analysis, we found patients in the GA group does not show lower rate of favorable functional outcome compared with the CS group (OR = 1.84, 95% CI: 1.17–2.89). And there was no significant difference in the rate of mortality between GA and CS groups during RCT subgroup analysis (OR = 0.74, 95% CI: 0.43–1.27).

**Conclusions:**

AIS patients performed endovascular treatment under GA compared with CS was associated with worse functional outcome and increased rate of mortality, but differences in worsened outcomes do not exist when one looks into the GA vs. CS RCTs. Moreover, these findings are mainly based on the retrospective studies and additional multi-center randomized controlled trials to definitively address these issues is warranted.

**Electronic supplementary material:**

The online version of this article (10.1186/s12871-019-0741-7) contains supplementary material, which is available to authorized users.

## Background

Acute ischemic stroke (AIS) is one of the leading causes of death and long-term disability. The common therapy for AIS patients with large-vessel occlusion is endovascular treatment [1]. During the endovascular treatment, there are two types of anesthesia /sedation which are commonly used to make the AIS patients immobile, including general anesthesia (GA) and conscious sedation (CS). However, the understanding of the impact of GA or CS on the outcomes of endovascular treatment remains controversial. Previous observational studies report worse outcomes from GA than that from CS during endovascular treatment [2]. By contrast, there were some new randomized trials found that functional independence or worse tissue is either no different in the patients who had GA [3–5]. While the available previous meta-analysis studies revealed superior neurological outcome with CS compared with GA [2, 6, 7]. But those meta-analysis studies were limited by the small sample size and the included studies were not comprehensive. Besides, there are updated and larger randomized clinical trials have conducted. In light of the continuing debate and limitations among these studies, a new and comprehensive meta-analysis study is warranted. We aim to compare the outcomes of AIS patients with GA and CS during the procedures.

## Methods

### Search strategy

This systematic review and meta-analysis was conducted in accordance with the PRISMA (Preferred Reporting Items for Systematic Reviews and Meta-Analyses) statement. All published articles were searched without language restricted in the PubMed, Embase, and Web of Knowledge databases through February 2019. Key words were identified and in various relevant combinations as follows: endovascular OR ‘fibrinolytic agents’ OR thromboembolism OR catheter OR transcatheter OR thrombolysis OR fibrinolysis OR recanalization OR embolectomy OR thrombectomy AND “intracranial embolism” OR thrombosis OR stroke AND “conscious sedation” OR “general anesthesia”. The updated literature search was conducted independently by two authors (L.L., T.-F. W.). All articles were retrieved and their references were manually screened to avoid missing out other relevant articles. If data could not be extracted in the original articles, we contacted the authors to obtain them.

### Study selection

The eligible studies were evaluated by two authors (T.-F.W., L.L.) independently. Disagreement was resolved by discussion, and if necessary, by a third reviewer (H.-S. C).The inclusion criteria were as follows: (1) studies evaluating the outcomes of general anesthesia versus conscious sedation during endovascular therapy among acute ischemic stroke patients; (2) studies reporting mortality or functional outcome using the modified Rankin scale (mRS) for the general anesthesia and conscious sedation groups; and (3) the effect estimates of studies could be extracted or calculated from the available data. We excluded those studies with unavailable data to calculate or extract effect estimates. The abstracts from meeting proceedings, duplicate reports, case reports, reviews, comments, or animal studies were also excluded.

### Data extraction

Two independent investigators (L.L., T.-F. W.) extracted following variables from the trials’ primary texts to ensure the reliability of the results, including the first author’s name, publication year, study period, country, inclusion criteria, exclusion criteria, outcomes, type of endovascular treatment, sample size, good outcomes (mRS scores ≤2 at 90 days) and other outcomes (including mortality, pneumonia, successful recanalization, symptomatic intracranial hemorrhage [sICH], asymptomatic intracranial hemorrhage [aICH], and vessel dissection or perforation) during the random trial. Disagreement was resolved by discussion, and if necessary, by a third reviewer (Z.-A. Z.).

### Quality assessment

Quality assessment of the studies was performed by two independent reviewers (L.L., T.-F. W.). We used the Newcastle-Ottawa Scale to assess the risk of selection, comparability and exposure or outcome for case-control and cohort studies, respectively. Eight items were included to assess the quality of studies with a 9-star system. The quality score ranges from 0 to 9 stars, we judged trials as a low-quality report study while the score is 0–3 stars, while a high quality study score is at least 7 stars. And the study with 4–6 stars was defined as a moderate quality study [8]. Moreover, we used Cochrane risk of bias tool to evaluate the quality of included RCTs [9].

### Statistical analysis

The outcomes in each included study, including favorable functional outcome (mRS scores ≤2 at 90 days), mortality, pneumonia, successful recanalization, sICH, aICH, and vessel dissection or perforation were extracted from primary trial results, succeeding secondary publications and their supplementary materials. For each study, odds ratios (ORs) were expressed with their 95% confidence intervals (CIs) for each outcome of interest and were calculated from patient numbers with each outcome categorized by different anesthesia type treatment. The random-effects meta-analysis (DerSimonian-Laird method) or fixed-effects meta-analysis (Mantel-Haenszel method) was used for pooling across studies and the statistical significance of pooled ORs and 95% CIs were determined with a Z test [10]. Moreover, which effects model we used was according to our heterogeneity test. To determine the degree of heterogeneity among the studies included in our meta-analysis, the I-squared (*I*^*2*^) statistic and the Cochran Q test were used [11], with *I*^*2*^ values less than 25% representing low heterogeneity, 25~50% representing moderate heterogeneity, and more than 75% representing high heterogeneity, respectively. When *I*^*2*^ values was less than 50%and the *P* value of the Q test was more than 0.1 among the studies included in the meta-analysis, the fixed-effects model was used for pooling across studies. While the *I*^*2*^ values was more than 50% and the *P* value of the Q test was less than 0.1, the random-effects model was used. Moreover, we performed sensitivity analysis by sequentially excluding each study that we have included to assess the stability of the results. To quantitate the publication bias across included studies, the Egger regression and Begg’s methods were used [12, 13]. All statistical tests were performed with STATA software version 12.0 (StataCorp, College Station, TX, USA). Statistical significance was based on a *P* value < 0.05 in all analyses.

## Results

### Characteristics of eligible studies

**Fig. 1:**
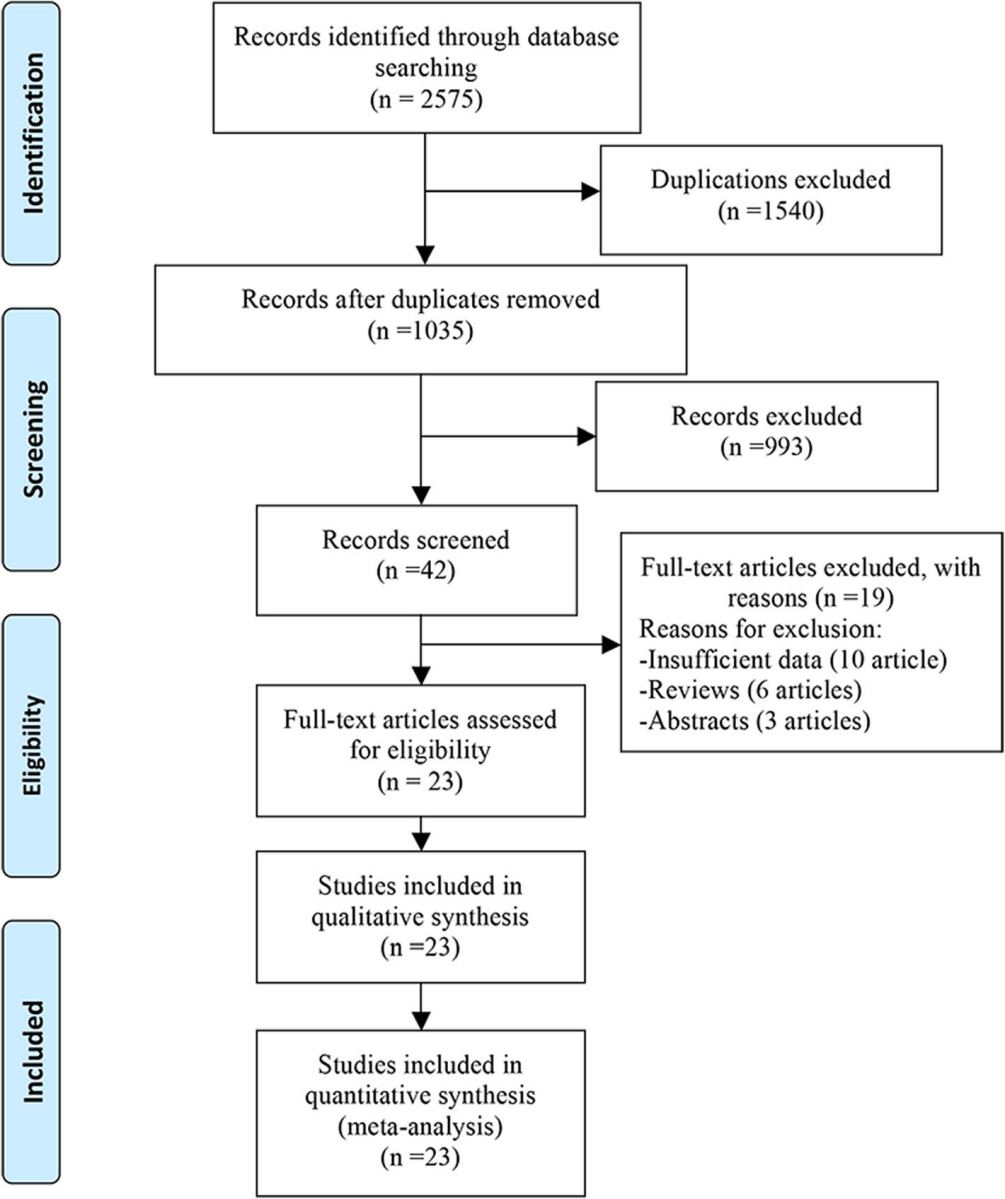
Flow diagram of study identification

The PRISMA flow diagram is shown in Fig. 1. In detail, total 2575 records were identified from PubMed, Web of Science and Embase. Of these, 1540 duplicated records and 993 unrelated records were excluded after reading the abstracts alone. Of the remaining 42 records, 19 records were excluded after further screen through full-text reading (10 records did not provide outcome of interest or available data, 6 reviews, and 3 abstracts). Finally, 23 records were eligible in our meta-analysis [3–5, 14–33], including 5 randomized controlled trials (RCTs) and 18 non-RCTs.

The characteristics of the included studies are presented in Table 1. These studies were published during 2010–2018. Ten of the 23 included studies were performed in the United States of America. The patients both in GA and CS groups have received endovascular treatment, such as IA /IV tPA, mechanical thrombectomy, stent and thromboaspiration. The baseline NIHSS scores of all patients were listed in Additional file 3: Table S1. All of the included studies were involved in the analysis of mortality rate. Majority of included studies provided information regarding the effect of GA versus CS on the incidence of mRS scores ≤2 at 90 days except for two studies [25, 28]. The results of the Newcastle-Ottawa Scale showed that 16 studies had high quality and 4 studies with a moderate quality, and no study had less than five pluses on the Newcastle-Ottawa scale (Table 1). The results of quality assessment for RCTs were listed in Additional file 3: Table S2.

**Table 1:** Characteristics of the studies included in the meta-analysis

Author	Study design	Study Period	Country	Inclusion Criteria	Exclusion Criteria	Outcomes	Type of Endovascular Treatment	Sample Size (GA/CS)	Methodological quality^a^
Abou-Chebl et al. (2010)	Retrospective cohort study	2005–2009	USA	Anterior circulation AIS	Posterior circulation strokes; no intervention was performed	mRS and mortality on day 90	IA/IV tPA, mechanical thrombectomy, angioplasty	428/552	5
Jumaa et al. (2010)	Retrospective cohort study	2006–2009	USA	Middle cerebral artery–M1 segment occlusion treated with endovascular therapy	Vertebrobasilar occlusions; internal carotid artery terminus occlusion; M2 occlusion	mRS and mortality on day 90	IA/IV tPA and mechanical thrombectomy	53/73	7
Nichols et al. (2010)	Post hoc analysis of IMS II trial	NA	USA	Anterior circulation strokes and underwent angiography and/or intervention	Data were not available before or after angiography	Recanalization, mRS and mortality on day 90	IA/IV tPA, low-energy ultrasound	26/49	7
Sugg et al. (2010)	Retrospective cohort study	2007–2009	USA	AIS and underwent endovascular treatment within 8 h from symptom onset	NA	Recanalization, mRS and mortality on day 90	Mechanical thrombectomy	9/57	5
Davis et al. (2012)	Retrospective cohort study	2003–2009	Canada	AIS and received endovascular treatment	Management could not be determined	mRS and mortality on day 90	IA tPA and mechanical thrombectomy	48/48	7
Hassan et al. (2012)	Retrospective cohort study	2006–2010	USA	AIS and received endovascular treatment	The infarct burden was greater than or equal to one third of the middle cerebral artery territory	mRS and in-hospital mortality	IV tPA, endovascular technique not specified	53/83	5
Langner et al. (2013)	Retrospective cohort study	2005–2010	Germany	AIS and treated with endovascular therapy	NA	mRS and mortality	IA tPA and mechanical thrombectomy	19/105	8
Abou-Chebl et al. (2014)	Post hoc analysis of NASA registry	NA	North America	AIS and received endovascular treatment within 6 h from symptom onset	NA	mRS and mortality on day 90	IV tPA, mechanical thrombectomy	196/85	7
John, S et al. (2014)	Retrospective cohort study	2008–2012	USA	Anterior circulation AIS and treated with endovascular therapy	Posterior circulation AIS	In-hospital mortality and mRS on day 30	IV tPA, mechanical thrombectomy	91/99	9
Li et al. (2014)	Retrospective cohort study	2006–2012	USA	AIS and received endovascular treatment	NA	mRS and mortality	IA tPA, mechanical thrombectomy	35/74	8
Abou-Chebl et al. (2015)	Post hoc analysis of IMS III trial	2006–2011	USA	AIS, received IV tPA within 3 h and received endovascular treatment	Large regions of clear hypodensity on CT scan	mRS and in-hospital mortality	IV/IA tPA and mechanical thrombectomy	147/269	9
McDonald J.S et al. (2015)	Retrospective cohort study	2006–2013	USA	AIS and received mechanical thrombectomy	Patients who underwent another invasive surgery	In-hospital mortality and complications	IV tPA and mechanical thrombectomy	507/507	7
Van Den Berg L.A. et al. (2015)	Retrospective cohort study	2002–2013	Netherlands	Anterior circulation AIS	Patients lack of information	mRS and mortality	IA tPA and mechanical thrombectomy	70/278	7
Just, C. et al. (2016)	Retrospective cohort study	2000–2013	Canada	Underwent neuro-interventional stroke procedure	Aneurysm repair, carotid stenting, extracranial-intracranial bypass	mRS and mortality on day 90 and 180	IA tPA and mechanical thrombectomy, thromboaspiration	42/67	8
Berkhemer OA. et al. (2016)	Post hoc analysis of MR CLEAN	2010–2014	Netherlands	AIS patients who received mechanical thrombectomy or intra-arterial thrombolysis	Cerebral hemorrhage, coagulation abnormalities	mRS and mortality on day 90 and 180	IA tPA and mechanical thrombectomy, thromboaspiration	79/137	9
Schönenberger S et al. (2016)	RCT	2014–2016	Germany	Severe AIS, NIHSS> 10	diagnostic imaging results did not clearly depict site of vessel occlusion	NIHSS after 24 h, mRS and mortality on day 90	IA tPA and angioplasty, mechanical thrombectomy, thromboaspiration	73/77	NA
Bekelis, K. et al. (2017)	Retrospective cohort study	2009–2013	USA	AIS patients undergoing mechanical thrombectomy	NA	mortality in hospital	IV tPA, thromboaspiration	441/733	6
Lowhagen Henden, P. et al. (2017)	RCT	2013–2016	Sweden	Anterior circulation AIS, NIHSS> 10	Occlusion of posterior cerebral circulation	mRS and mortality on day 90 and 180	IV tPA, mechanical thrombectomy	45/45	NA
Slezak, A.et al. (2017)	Prospective study	2010–2015	Switzerland	Anterior circulation AIS	NA	mRS and mortality on day 90	IV tPA, mechanical thrombectomy	266/135	7
Simonsen, C. Z. et al. (2018)	RCT	2015–2017	Denmark	Anterior circulation AIS	Glasgow Coma Scale score < 9 or premorbid mRS score > 2	mRS and mortality on day 90 and 180	IA tPA, angioplasty, mechanical thrombectomy	65/63	NA
Peng et al. (2018)	Prospective study	2015.01–2015.08	China	Anterior circulation AIS	DWI lesion volume > 50 mL	Rates of successful recanalization and mRS on day 90	Interventional treatment with Solitaire	44/105	8
Omer F. Eker et al. (2018)	Post hoc analysis SWIFT PRIME trial	2013–2015	USA and Europe	Anterior circulation AIS	Subject who is contraindicated to IV t-PA	Rates of successful recanalization and mRS on day 90	Teated with Solitaire RevascularizationDevice and IV-tPA	32/65	8
Shanet et al. (2018)	Retrospective cohort study	2014–2016	China	Anterior circulation AIS	Received intra-arterial thrombolysis alone, with concomitant aneurysm or arteriovenous malformation	mRS and mortality on day 90	NA	114/114	8

^a^Newcastle Ottawa scale was designed to assess the quality of non-randomized studies. *GA* general anesthesia, *CS* conscious sedation, *AIS* acute ischemic stroke, *IA* intra-arterial, *IV* intra-venous, *tPA* tissue plasminogen activator, *mRS* modified Rankin Score, *NA* not available, *RCT* randomized controlled trial, *NIHSS* National Institute of Health Stroke Scale

### Outcomes

**Fig. 2:**
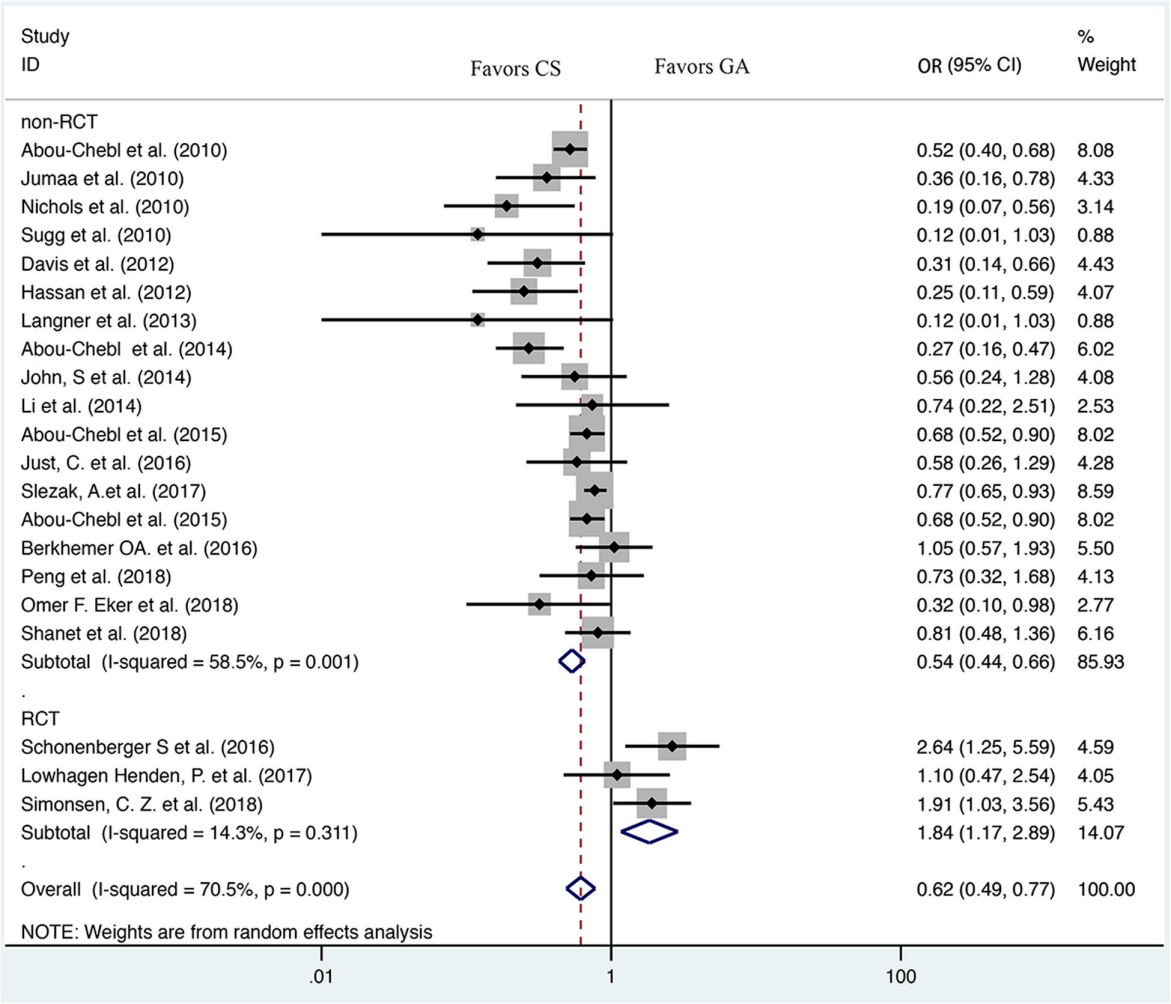
Forest plot of meta-analysis results for good functional outcome (mRS ≤ 2). OR, odds ratio; CI, confidence interval

**Fig. 3:**
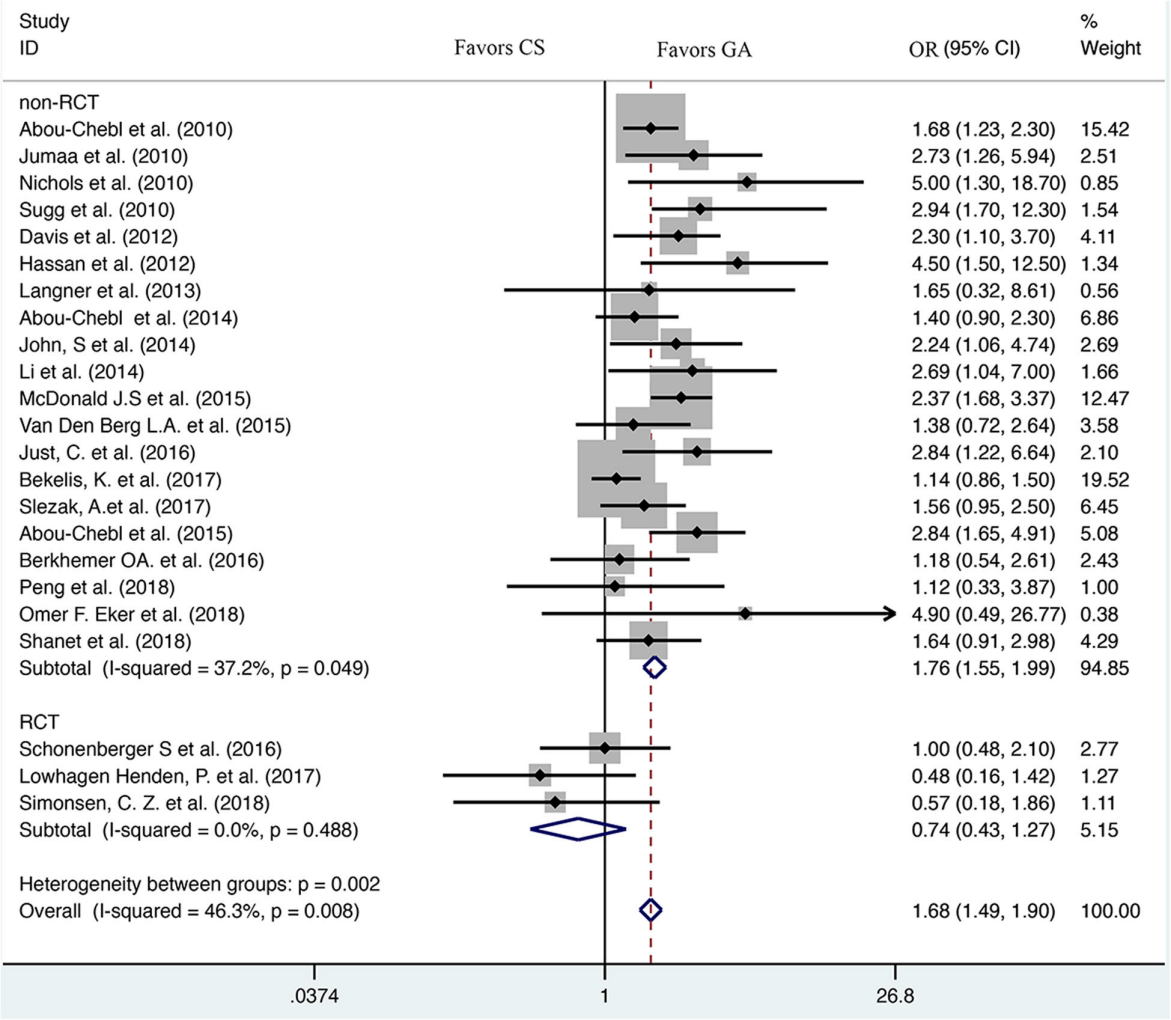
Forest plot of meta-analysis results for the risk of mortality. OR, odds ratio; CI, confidence interval

Six thousand seven hundred three patients were included in this meta-analysis in total, including 3820 patients in CS group and 2883 patients in GA group. The results of our meta-analysis suggested that GA patients have lower odds of favorable functional outcome (mRS scores ≤2) compared with CS patients (OR = 0.62, 95% CI: 0.49–0.77) (Fig. 2, Table 2). Moreover, GA was associated with a statistically significant higher risk of mortality (OR = 1.68, 95% CI: 1.49–1.90) (Fig. 3, Table 2), pneumonia (OR = 1.78, 95% CI: 1.40–2.26) (Table 2) and sICH (OR = 1.64, 95% CI: 1.13–2.37) (Table 2). However, there were no significant differences in the rate of recanalization (OR = 1.07, 95% CI: 0.89–1.28) (Table 2), vessel dissection or perforation (OR = 1.00, 95% CI: 0.98–1.03) (Table 2), aICH (OR = 1.19, 95% CI: 0.96–1.47) (Table 2) between the two groups.

**Table 2:** Summary of meta-analysis results

Groups	Test of association				Heterogeneity		
	OR [95%CI]	*p* value	Model	Z	Χ^2^	*p* value	*I*^2^ (%)
mRS score (0–2)	0.62 [0.49–0.77]	< 0.001	RE	4.16	67.83	< 0.001	70.5%
Mortality	1.68 [1.49–1.90]	< 0.001	FE	8.28	40.98	0.008	46.3%
Successful recanalization	1.07 [0.89–1.28]	0.943	FE	0.47	26.35	0.023	46.9%
Vessel dissection or perforation	1.00 [0.98–1.03]	0.010	FE	0.19	11.21	0.34	10.8%
sICH	1.64 [1.13–2.37]	0.010	RE	2.59	31.63	< 0.001	68.4%
aICH	1.19 [0.96–1.47]	0.116	FE	1.57	3.36	0.644	0.0%
Pneumonia	1.78 [1.40–2.26]	< 0.001	FE	4.67	7.95	0.539	0.0%

*OR* odds ratio, *CI* confidence interval, *mRS* modified Rankin Score, *RE* random effects, *FE* fixed effects, *sICH* symptomatic intracranial hemorrhage, *aICH* asymptomatic intracranial hemorrhage

While in the subgroup analysis, the rate of favorable functional outcome (mRS scores ≤2) in non-RCT subgroup also showed a statistically significant lower between CS and GA group (OR = 0.54, 95% CI: 0.44–0.66) but not in RCT subgroup (OR = 1.84, 95% CI: 1.17–2.89) (Fig. 2). Besides, GA was associated with significantly higher rate of mortality than CS in non-RCT subgroup (OR = 1.76, 95% CI: 1.55–1.99), but there was no significant difference in the rate of mortality between GA and CS groups during RCT subgroup analysis (OR = 0.74, 95% CI: 0.43–1.27) (Fig. 3).

Moreover, we have conducted an additional separate meta-analysis for high quality studies (quality scores ≥7 and RCTs). We found the use of GA was also associated with poorer neurologic outcome at 90 days (OR = 0.64, 95% CI: 0.50–0.83) (Additional file 1: Figure S1) and higher mortality (OR = 1.83, 95% CI: 1.57–2.14) (Additional file 2: Figure S2) compared with CS.

### Heterogeneity and sensitivity analysis

Obvious between-study heterogeneity (*I*^*2*^ values more than 50%) was found for the following outcomes: good functional outcome (mRS scores ≤2) (*I*^*2*^ = 70.5%) and sICH (*I*^*2*^ = 68.4%). While no obvious heterogeneity (*I*^*2*^ values less than 50%) was detected in death at 90 days (*I*^*2*^ = 46.3%), successful recanalization (*I*^*2*^ = 46.9%), pneumonia (*I*^*2*^ = 0.0%), vessel dissection or perforation (*I*^*2*^ = 0.0%) and aICH (*I*^*2*^ = 0.0%).

Each study was removed sequentially to verify the effect of each individual study in our results. There are no other important changes in our pooled OR value after excluded any study. Therefore, our results were reliable (data not shown).

### Publication bias

Assessment of publication bias was performed by both Egger’s and Begg’s methods in our meta-analysis. And the results showed that there were no significant publication bias among the included studies (Begg’s test: *P* = 0.780, Egger’s test: *P* = 0.352).

## Discussion

Using a comprehensive meta-analysis, we identified the worse functional outcome and higher rate of mortality among AIS patients as they received GA during endovascular treatment. Besides, we found patients in the GA group are associated with higher rate of sICH and often had more pneumonia. While no clinically meaningful differences in recanalization rate, aICH, vessel dissection or perforation were seen between patients under CS and GA. In contrast, in the RCT subgroup analysis, the difference of worse functional outcome do not exist when one looks into the GA vs. CS.

The exact reasons that why the CS group showed lower rate of mortality and better functional outcome may be multifactorial. It was well known that the purpose of general anesthesia is to decrease intraprocedural patient movement. If the patients are awake during endovascular treatment, they would move casually and be agitated during treatment, which affects Digital Subtraction Angiography images and leads to wire perforation and may result in significant vascular injury and intracranial hemorrhage. In contrast to this theory, recent meta-analysis study did not demonstrate that CS was associated with higher rate of intracranial hemorrhage than GA [34]. Moreover, in our results, we found that patients in the GA group has a higher rate of sICH. Additionally, GA has been associated with significantly higher treatment costs ($46,444 VS $30,350) [24]. And GA may limit the ability of the interventionalist to assess neurological status during the procedure. Thus, these findings do not support GA as a safer and lower cost approach for endovascular thrombectomy treatment. Moreover, as one of main factors, delay of treatment was also commonly concerned. As the post hoc analysis of MR CLEAN showed, a longer delay for patients in the GA group was observed. Intra-arterial therapy was initiated sooner after symptom onset in patients treated with non-GA as compared with GA [28]. Recently, a meta-analysis from 5 randomized controlled trials also revealed that 1 hour of delay in door-to-puncture times could reduce 19% likelihood of regaining functional independence [35]. Thus, it is reasonable to assume that treatment delays during GA may result in a disadvantage. However, the detail of door-to-puncture times among all the included studies in this meta-analysis could not be obtained completely. Therefore, future trials should study the effect of the time delay from hospital admission to vessel puncture on outcomes and its possible interaction with the type of anesthesia.

On the other hand, the poorer outcomes in GA may related with the poorer clinical status in patients who were chosen and different anesthetic agents were used for the procedure under GA. As previous studies reported, inhaled anesthetic agents were generally used for GA, which are often associated with hemodynamic disturbance, including rapid blood pressure fluctuations and lower blood pressure, which would lead to decrease of cerebral bloodflow and exacerbate ischemic injury [36, 37]. For example, Reich et al. have revealed that using propofol and the induction dose of fentanyl may cause post induction hypotension [38]. Both in the AnStroke and GOLIATH trials, blood pressure was lower in the GA group [3, 4]. In contrast, using dexmedetomidine for patients undergoing endovascular stroke treatment, which could stabilize blood pressure and prevent hypotension with induction, and improve outcomes consequently [39]. Besides, a good outcome shows an association with a higher pre-anesthesia blood pressure, while the pre-anesthesia blood pressure was negatively correlated with GA use [18, 21]. Thus, those studies indicated that the deleterious effects of GA may due to the changes of blood pressure. It is conceivable that we should pay more attention to evaluating the effects of blood pressure on outcomes and the interaction between blood pressure and the type of anesthesia should also be observed.

Different anesthetic agents may show protective or harmful effects on ischemic brain, but there are no conclusive data about the neuroprotective properties of anesthetic agents to help recommending an anesthetic agent [40]. Numerous preclinical studies indicate that isoflurane shows neuroprotective effects in ischemic preconditioning and postconditioning by alleviating glutamate excitotoxicity and opening of potassium channels [41, 42]. Besides, intravenous propofol has also been suggested as a neuroprotective agent on ischemic stroke by many molecular pathways [43]. However, these findings were just demonstrated in nonhuman primate studies. A retrospective study for endovascular management of AIS suggested that volatile anesthetics are superior to intravenous agents, but this finding should be validated by a larger randomized controlled trial [44]. Thus, to minimize the confounding effects of different drugs during endovascular treatment, the same anesthetic agent should be used as both a general anesthetic and a sedative. Among the included studies in this meta-analysis, the specific data on the type of anesthetic agent used in the GA or CS patients are unavailable in most included studies, but the GOLIATH trial has used the propofol as both a general anesthetic and a sedative in the CS group [3].

Actually, the choice of GA or CS for a given AIS patient in clinical practice, which were mainly decided by the patient’s physical status. For instance, the AIS patients with underlying medical comorbidities or stroke severity may be performed with GA as “medically indicated” [18]. Moreover, as Abou-Chebl et al. have stated that a major weakness of the retrospective study was that the association between GA and poor outcomes may be due to the AIS patients with aphasia or who were unable to follow commands and necessitated GA [24]. In non-randomized studies, the choice of GA or CS for a given AIS patient was most likely due to either technical concerns (difficulty of interventional procedure) or safety concerns (airway patency). Consequently, in the subgroup analysis of this study, we have revealed inconsistent findings between the randomized and nonrandomized studies. These findings were also consistent with the previous meta-analyses by Jing R. and his colleagues, but our study have included more studies [7]. Taken together, the “medically indicated” highlights the problem of bias and may explain the reason that why the randomized and nonrandomized studies show marked discrepancy in results.

We must acknowledge that this study has several limitations. The design of included studies were various and the choice of CS or GA for a given AIS patient was not randomized for most of included studies. Thus, we conducted the subgroup analysis according to the design type of the included trials and the subgroup analysis showed inconsistent results, but we could not find the exact reasons for this discrepancy due to the lack of some of essential reported data (eg, the type and dose of anesthetic agents used, stroke location, time to treatment, baseline NIHSS scores). Although, parts of included studies have presented the baseline NIHSS scores for patients in GA and CS groups, we could not pool all the baseline NIHSS scores of patients for all included trials, because these studies presented data in different forms [mean (SD) or mean (IQR)] and the individual data were unobtainable. Moreover, in clinical practice, there was a lower rate of anterior circulation occlusions in the GA group than in the CS group. As a meta-analysis of individual patients with anterior circulation showed, outcomes were significantly better for patients who did not receive GA versus those who received GA [34]. However, we could not conduct the subgroup analyses according to those factors in the present study for relatively small or incomparable number of available studies.

## Conclusions

In summary, the pooled data from this meta-analysis indicated that performing endovascular treatment under GA compared with CS was associated with worse functional outcome and increased rate of mortality. However, in the RCT subgroup analysis, differences in worsened outcomes do not exist between GA and CS group. Moreover, these findings are mainly based on the retrospective studies that did not randomize patients by anesthesia type. Thus, additional multi-center RCTs to definitively address these issues is warranted. In addition, to understand the exact reasons which cause the differences between the GA and CS when AIS patients are performed endovascular treatment, future studies should consider the underlying confounding factors (eg, door-to-puncture times, baseline NIHSS scores, blood pressure level).

## Additional files

Additional file 1: **Figure S1.** Forest plot of meta-analysis results for good functional outcome (mRS ≤ 2) among the high quality studies. OR, odds ratio; CI, confidence interval. (TIF 3752 kb)

Additional file 2: **Figure S2.** Forest plot of meta-analysis results for the risk of mortality among the high quality studies. OR, odds ratio; CI, confidence interval. (TIF 3976 kb)

Additional file 3: **Table S1.** The baseline NIHSS scores of patients in each included trials. **Table S2.** Assessment of the methodological quality of included randomized trials using the Cochrane Collaboration’s Tool. (DOCX 24 kb)

## Abbreviations

aICH: Asymptomatic intracranial hemorrhage
AIS: Acute ischemic stroke
CI: Confidence interval
CS: Conscious sedation
FE: Fixed effects
GA: General anesthesia
IA: Intra-arterial
IV: Intra-venous
mRS: Modified Rankin scale
NIHSS: National Institute of Health Stroke Scale
OR: Odds ratio
PRISMA: Preferred Reporting Items for Systematic Reviews and Meta-Analyses
RCT: Randomized controlled trial
RE: Random effects
sICH: Symptomatic intracranial hemorrhage
tPA: Tissue plasminogen activator
