# Supplementary figures and images for: Outcomes of general anesthesia versus conscious sedation for Stroke undergoing endovascular treatment: a meta-analysis
Source: BMC Anesthesiol. 2019 May 10;19:69. doi: 10.1186/s12871-019-0741-7 (PMC6511209; doi:10.1186/s12871-019-0741-7)

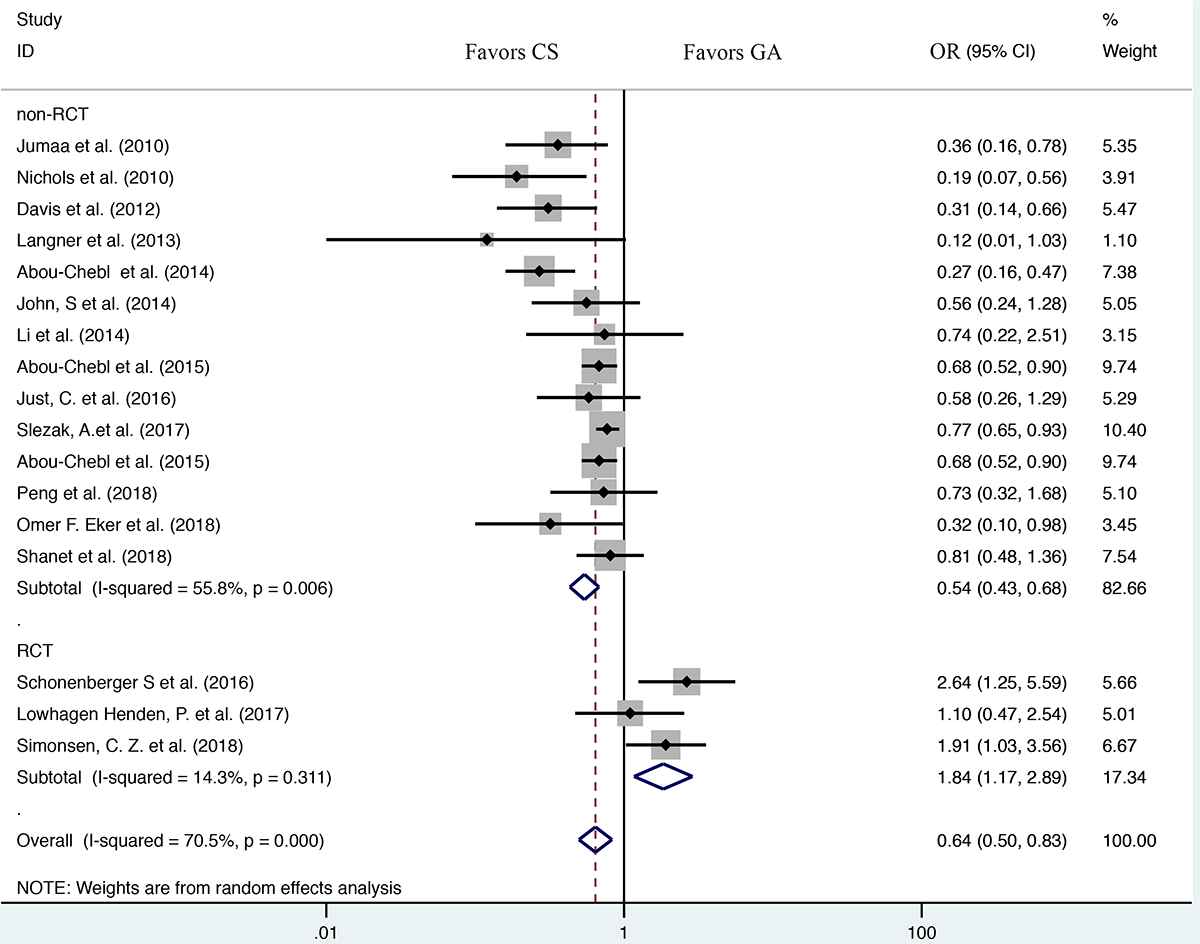

Supplement: Supplementary file 1 — Figure S1. Forest plot of meta-analysis results for good functional outcome (mRS ≤ 2) among the high quality studies. OR, odds ratio; CI, confidence interval. (TIF 3752 kb) [file 12871_2019_741_MOESM1_ESM.tif]

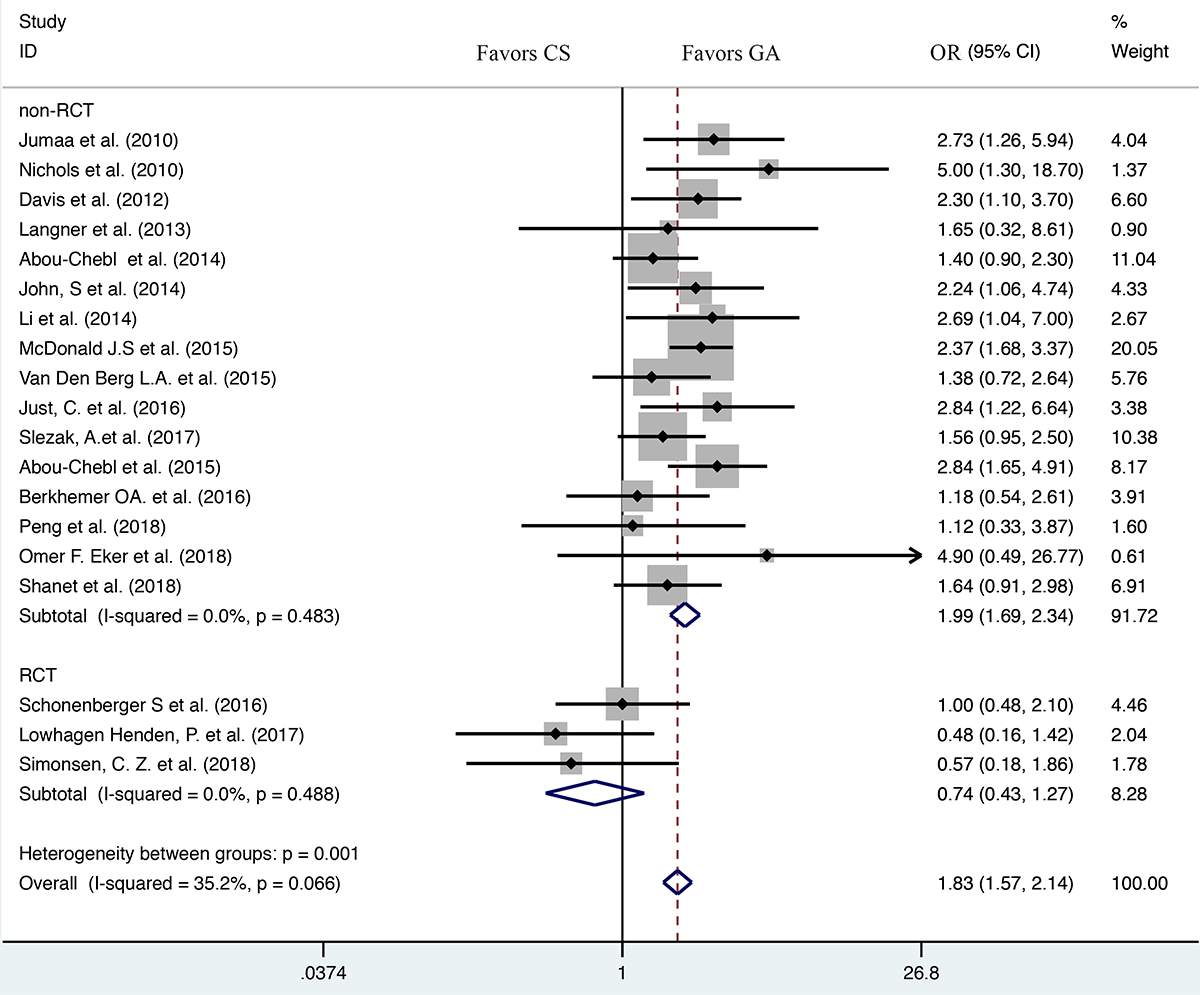

Supplement: Supplementary file 2 — Figure S2. Forest plot of meta-analysis results for the risk of mortality among the high quality studies. OR, odds ratio; CI, confidence interval. (TIF 3976 kb) [file 12871_2019_741_MOESM2_ESM.tif]
