# Supplementary material for: Outcomes of general anesthesia versus conscious sedation for Stroke undergoing endovascular treatment: a meta-analysis
Source: BMC Anesthesiol. 2019 May 10;19:69. doi: 10.1186/s12871-019-0741-7 (PMC6511209; doi:10.1186/s12871-019-0741-7)
Supplement: Supplementary file 3 — Table S1. The baseline NIHSS scores of patients in each included trials. Table S2. Assessment of the methodological quality of included randomized trials using the Cochrane Collaboration’s Tool. (DOCX 24 kb) [file 12871_2019_741_MOESM3_ESM.docx]

**Outcomes of** **General Anesthesia** **versus Conscious Sedation for Stroke Undergoing** **Endovascular Treatment:** **A Meta-analysis**

**Supplementary Online Content**

**Table S1**. The baseline NIHSS scores of patients in each included trials.

**Table S2.** Assessment of the methodological quality of included randomized trials using the Cochrane Collaboration’s Tool

**Table S1**. The baseline NIHSS scores of patients in each included trials.

| **Author** | NIHSS scores | | P Value |
| --- | --- | --- | --- |
|  |  |  |  |
|  | GA | CS |  |
| Abou-Chebl et al. (2010) | 15±5 | 18±6 | 0.0001 |
| Jumaa et al. (2010) | 17.6 (14–22) | 15.1 (12–18) | 0.004 |
| Nichols et al. (2010) | NA | NA | NA |
| Sugg et al. (2010) | 17.1 ± 7.4 | 28.3 ± 5.0 | <0.001 |
| Davis et al. (2012) | 19.5 (9) | 16 (9.5) | 0.03 |
| Hassan et al. (2012) | NA | NA | NA |
| Langner et al. (2013) | NA | NA | NA |
| Abou-Chebl et al. (2014) | 18.9±6.9 | 16.4±5.8 | 0.002 |
| John, S et al. (2014) | 16.3±6.8 | 15.4±6.4 | 0.364 |
| Li et al. (2014) | 18 (7) | 15 (6) | 0.059 |
| Abou-Chebl et al. (2015) | 18 (7-40) | 16 (7-29) | NA |
| McDonald J.S et al. (2015) | NA | NA | NA |
| Van Den Berg L.A. et al. (2015) | 16 (5) | 15 (7) | 0.76 |
| Just, C. et al. (2016) | 13.36 | 12.88 | 0.77 |
| Berkhemer OA. et al. (2016) | 18 (15–21) | 17 (14–21) | NA |
| Schönenberger S et al. (2016) | 16.8 (3.9) | 17.2 (3.7) | 0.52 |
| Bekelis, K. et al. (2017) | NA | NA | NA |
| Lowhagen Henden, P. et al. (2017) | 20 (15.5–23) | 17 (14–20.5) | NA |
| Slezak, A.et al. (2017) | 13.1 (5.7) | 17.2 (6.6) | < 0.001 |
| Simonsen, C. Z. et al. (2018) | 18 (13-21) | 17 (15-21) | 0.84 |
| Peng et al. (2018) | 16 (12-19) | 17 (12-21.5) | 0.59 |
| Omer F. Eker et al. (2018) | 16.8 ± 4.8 | 16.1 ± 4.0 | 0.44 |
| Shanet et al. (2018) | 16 (11–21) | 16 (12–20) | 1.00 |

NIHSS, National Institute of Health Stroke Scale; GA, general anesthesia; CS, conscious sedation; NA, not available; RCT, randomized controlled trial.

**Table S2.** Assessment of the methodological quality of included randomized trials using the Cochrane Collaboration’s Tool

| **Trials** | Sequence generation | Allocation concealment | Blinding of participants, personnel and outcome assessors | Incomplete outcome data | Selective outcome reporting | Other sources of bias |
| --- | --- | --- | --- | --- | --- | --- |
| Schonenberger S et al. (2016) | Low | Low | High | Low | Low | Low |
| Lowhagen Henden, P. et al. (2017) | Low | Low | High | Low | Low | Low |
| Simonsen, C. Z. et al. (2018) | Low | Low | High | Low | Low | Low |
